# Classifying nursing organization in wards in Norwegian hospitals: self-identification versus observation

**DOI:** 10.1186/1472-6955-9-3

**Published:** 2010-02-09

**Authors:** Ingeborg S Sjetne, Jon Helgeland, Knut Stavem

**Affiliations:** 1 Norwegian Knowledge Centre for the Health Services, PO Box 7004, St Olavs plass, N-0130 Oslo, Norway; 2 Institute of Nursing and Health Sciences, University of Oslo, Norway; 3 Medical Division, Akershus University Hospital, Lørenskog, Norway; 4 Helse Sør-Øst Health Services Research Centre, Lørenskog, Norway; 5 Faculty of Medicine, University of Oslo, Oslo, Norway

## Abstract

**Background:**

The organization of nursing services could be important to the quality of patient care and staff satisfaction. However, there is no universally accepted nomenclature for this organization. The objective of the current study was to classify general hospital wards based on data describing organizational practice reported by the ward nurse managers, and then to compare this classification with the name used in the wards to identify the organizational model (self-identification).

**Methods:**

In a cross-sectional postal survey, 93 ward nurse managers in Norwegian hospitals responded to questions about nursing organization in their wards, and what they called their organizational models. K-means cluster analysis was used to classify the wards according to the pattern of activities attributed to the different nursing roles and discriminant analysis was used to interpret the solutions. Cross-tabulation was used to validate the solutions and to compare the classification obtained from the cluster analysis with that obtained by self-identification. The bootstrapping technique was used to assess the generalizability of the cluster solution.

**Results:**

The cluster analyses produced two alternative solutions using two and three clusters, respectively. The three-cluster solution was considered to be the best representation of the organizational models: 32 team leader-dominated wards, 23 primary nurse-dominated wards and 38 wards with a hybrid or mixed organization. There was moderate correspondence between the three-cluster solution and the models obtained by self-identification. Cross-tabulation supported the empirical classification as being representative for variations in nursing service organization. Ninety-four per cent of the bootstrap replications showed the same pattern as the cluster solution in the study sample.

**Conclusions:**

A meaningful classification of wards was achieved through an empirical cluster solution; this was, however, only moderately consistent with the self-identification. This empirical classification is an objective approach to variable construction and can be generally applied across Norwegian hospitals. The classification procedure used in the study could be developed into a standardized method for classifying hospital wards across health systems and over time.

## Background

Nursing services in hospital wards are structured and organized in different ways, with corresponding variations in the organization of the workforce and care processes. The choice of organizational model is an important element of service provision and influences the day-to-day running of a ward.

The organization of nursing services is often described using three generic models: (1)*Functional nursing*: tasks are allocated by similar principles to those of production lines, e.g. one registered nurse (RN) is responsible for infusion therapy for all patients, whereas another dresses wounds; (2)*Team nursing*: a small group of nurses with different qualifications are responsible for the care of several patients, so reducing the number of interpersonal contacts and clarifying the lines of responsibility. An RN is the team leader, supervising the work of less qualified personnel; (3)*Primary nursing: *one RN carries out all the care needed for a few patients. Primary nursing is conducive to the idea of holistic care and is assumed to lead to a good work environment [1-4]. In everyday practice the organization of nursing services is customized to fit local conditions such as staffing, workload, ward size, interdisciplinary cooperation, working hours and regulatory environment. Accordingly, the organization of two wards could have different theoretical bases and names but in practice the distinction may not be clear cut. It has been suggested, therefore, that self-identification (i.e. model identified by staff on ward) should be avoided as a way of describing nursing organization [3].

A valid classification of nursing organization would be useful to identify groups of wards that can be compared, or to be used as a covariate in multivariate analysis. Explicit and verifiable descriptions would also facilitate the review of studies of complex interventions, e.g. nursing service organization [5]. A recent review of studies on hospital organization recommended the development of standardized instruments for collection of primary data [6].

This paper presents a study of wards in Norwegian hospitals using a classification procedure that could be developed to have universal applicability and be useful in nursing research.

Researchers have used different approaches to classify nursing service organization at the ward level. In a study of staff nurses' perceptions of staffing adequacy, Kramer and Schmalenberg listed six organizational models for collecting staff nurses' descriptions of the organizational models in their units [7]: (1) new team, (2) total patient care, (3) modified primary, (4) old team, (5) true primary and (6) varying from day to day. Aiken and Patrician used the Revised Nursing Work Index questionnaire to study the nursing practice environments [8]. They asked staff nurses how much they agreed with the following statements: 'Team nursing is the nursing delivery system', 'Total patient care is the delivery system' and 'Primary nursing is the delivery system.' In a study of nursing shortage, Seago *et al*. [9] used three different organizational models: (1) primary/total, (2) team/functional and (3) modular/case management. A recent review by Kane *et al*. [10] of studies on nurse staffing and quality of patient care named five organizational models: (1) patient-focused care, (2) primary nursing, (3) total nursing care, (4) team nursing and (5) functional nursing. In addition, a study by Adams *et al*. [1] of aspects such as job satisfaction and within-ward cooperation identified three organizational models: (1) devolved, (2) two tier and (3) centralized. There is apparently a lack of consensus about the classification and nomenclature of nursing organization at the ward level [11].

Minnick *et al*. [3] suggested that the lack of conclusive research findings with regard to the strengths and weaknesses of different organizational models is a consequence of indistinct classifications; there is a need for knowledge on this topic to guide decisions in nursing administration re work force deployment, with the aim of improving patient outcomes and nurses' work conditions. The predicted health personnel shortage [12] could lead to modifications of existing organizational models [13] with a consequent increase in the need for knowledge. Previous research has indicated that higher numbers of RNs on the staff are associated with better patient outcomes [10] and human resource management practices in hospitals (including the extent of team working) are associated with hospital mortality [14].

Wards in Norwegian hospitals usually have the following names for their organizational model: primary nursing, patient-responsible nursing, modified primary nursing, team nursing and group nursing. Only licensed nursing personnel are employed in Norwegian hospitals, RNs with 3 years of college education being in the majority; the rest are licensed practical nurses with vocational qualifications.

For a nursing administration study [15], a simple and valid classification system was needed to categorize models of nursing service organization at the ward level. Our expectation was that self-identification of organizational models would only to some degree correspond with reported ward data describing activity patterns on the wards. Consequently, the aims of this study were: (1) to construct a variable to classify the organizational models in a sample of hospital wards, based on reported data about ward practice; and (2) to compare this classification with the self-identification reported by ward nurse managers.

## Methods

### Study design and sample

The study was part of a cross-sectional postal survey of hospital wards in 2005. Three groups of participants in each ward responded to the questionnaires: patients reported their experiences, staff nurses reported their perceptions of the practice environment and ward nurse managers provided information about overall ward characteristics. The hospital ward was the primary sampling unit. The sample size was set at 100, based on considerations of statistical power relating to the patient experience questionnaire [16] and assuming a 10% drop-out rate. The current study used the data collected from the ward nurse managers.

The study population consisted of 243 wards, with 18 or more beds in public general hospitals in Norway that performed acute, somatic care for adult patients 24 hours a day, 7 days a week. Maternity wards and wards with intensive or intermediate care beds were excluded. Background data were collected about hospital and ward size, geographical region and type of care provided. Ward managers of 156 wards consented to inclusion in the study. The initial random sample of 100 wards was reduced to 93 after reassessment of inclusion criteria and a check for completeness of data.

### Questionnaire

The questionnaire contained items that had been used in previous studies. Of the studies used, the authors of the earliest study [17] initially selected items based on a literature review of discriminating features of organizational models. They tested the questionnaire in a sample of ward nurse managers and suggested modifications. In four wards two nurse leaders completed the questionnaire, and the agreement in responses among these four pairs was excellent. Another study [18] used a similar questionnaire and compared staff nurses' responses to the questionnaire with the ward nurse managers' description of ward practice, reporting agreement re categorizations in 28 of 32 wards [18]. This approach has since been modified and used in other studies [1,19].

In the current study we translated and adapted questionnaire items used in the above studies for a Norwegian context. Nurse managers and nurse researchers took part in the final item selection and adaptation, aiming for a short and relevant questionnaire.

The questionnaire asked ward nurse managers which of six RN roles were usually responsible for seven important activities (see Additional file 1A). They were also asked what they called the organizational model (self-identification) and to provide supplementary data about medication administration, patient and work allocation, and scheduling of shifts on the ward (see Additional file 1B).

### Data analysis

#### Variable coding

Each RN role in each ward was scored with the number of activities attributed to it, ranging from 0 (no activities) to 7 (all activities listed) (see Additional file 1A).

The following were the six RN roles: (1) Any RN dealing with the patient, (2) Any RN in the patient's team, (3) Team leader, (4) Primary nurse, (5) RN in charge of shift and (6) Ward nurse manager.

The following were the seven activities: (1)Write and revise the nursing plan, (2) Report follow-up in the nursing plan, (3) Take part in the pre-round meeting with the doctors, (4) Accompany doctors on rounds, (5) Liaise with other professionals in the hospital, (6) Contact patients' relatives and (7) Plan patients' discharge.

For example, a score of 7 for the team leader role indicated that team leaders usually performed all the activities listed.

### Statistical analyses

To check for potential sampling bias we used the *t*-test and χ^2 ^test to compare background data for the study sample with data from the wards where consent was not given.

K-means cluster analysis of the RN role scores was used to classify the wards. This method requires that the researcher specify the number of clusters. Two-, three- and four-cluster solutions were tried out. Interpretation of the clusters and assessment of their separation were based on a profile diagram and discriminant analysis. The resulting discriminant functions give a low-dimensional representation of the multidimensional arrangement of data points and clusters [20]. The bootstrapping technique [21] was used to assess the generalizability of the clustering, and the minimum number of wards per cluster was set at 20 in the replications.

Cross-tabulation was used to examine correspondence between self-identification and the proposed clusters, and to assess criterion validity by comparing the supplementary data among the proposed clusters. Fisher's exact test was used to compare pairs of clusters. Because of multiple testing a 1% significance level was chosen.

We expected there to be some degree of correspondence between self-identification and cluster membership. Wards with extensive responsibilities for the primary nurse were expected to have a larger proportion of the patients allocated to a named/primary nurse, and for administration of oral medication and work allocation to be performed by primary nurses rather than team leaders, in contrast to wards with a prominent team leader. Furthermore, scheduling of shifts was expected to prioritize continuity of individual RN-patient relationships in primary nurse-dominated wards.

The software that we used was SPSS version 15 (SPSS Inc., Chicago, IL) for all analyses except for the bootstrapping, for which the R software was used http://www.R-project.org.

The Regional Committee for Medical Research Ethics and the Ombudsman for privacy in research at the Norwegian Social Science Data Service approved the study.

## Results

### Descriptive statistics for the wards

There were no statistically significant differences in geographical region, type of care provided or bed capacity between the sampled wards and the wards on which consent was not given. However, the latter were part of larger hospitals (Table 1).

**Table 1:** Sample characteristics and comparison to non-consenting wards.

	**Wards in the sample**		**Wards that did not consent to participate**		***p***
			
	***n***	**%**	***n***	**%**	
					
Geographical region					0.527^a^
Central Norway	20	22	14	16	
Northern Norway	7	8	12	14	
Southern Norway	17	18	15	17	
Western Norway	19	20	22	25	
Eastern Norway	30	32	24	28	
Total	93	100	87	100	
					
Type of care provided					
Surgical	23	25	34	39	0.282^a^
Medical	45	48	35	40	
Orthopaedics	13	14	7	8	
Neurology	5	5	5	6	
Mixed and gynaecology	7	8	6	7	
Total	93	100	87	100	
					
	Mean	SD	Mean	SD	
					
Hospital bed capacity	312	226	403	314	0.027^b^
					
Ward bed capacity	25.2	4.9	25.5	4.2	0.694^b^

^a^χ^2 ^test, ^b ^*t*-test

### Classification of hospital wards

The two-cluster solution produced one cluster of 70 wards and one of 23 wards, whereas the three-cluster solution yielded cluster A with 32 wards, cluster B with 23 wards and cluster C with 38 wards. Together, clusters A and C coincided with the largest cluster of the two-cluster solution. In the four-cluster solution, cluster sizes varied from 7 to 34.

### Interpretation of the clusters

**Figure 1:**
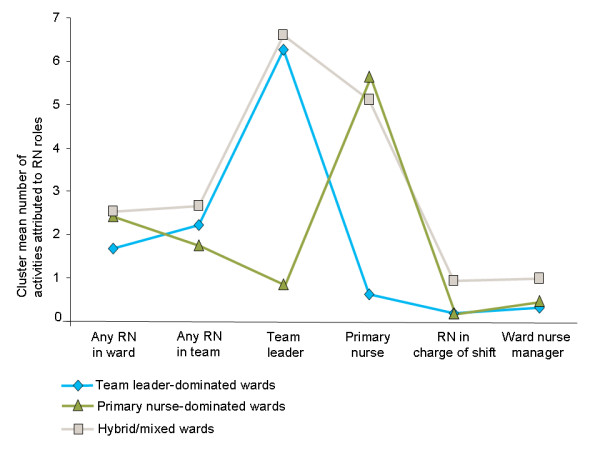
**Cluster profile diagrams on variables used in the clustering procedure: three-cluster solution**.

In the three-cluster solution, cluster A had a high score for 'Team leader' and a low score for 'Primary nurse' (Figure 1). This cluster was interpreted as team leader-dominated (TLD). Cluster B scored high on 'Primary nurse' and a low on 'Team leader', and was interpreted as primary nurse-dominated (PND). Cluster C scored high on both 'Team leader' and 'Primary nurse'.

Based on the discriminant function coefficients (Additional file 2), we interpreted the first discriminant function as a measure of team orientation and the second as a measure of individual nurse orientation. The 'Team leader' and 'Primary nurse' scores had the highest coefficients in the first and second functions, respectively. The scores for 'Any RN in team' and 'Any RN in ward' also contributed to the cluster discrimination.

**Figure 2:**
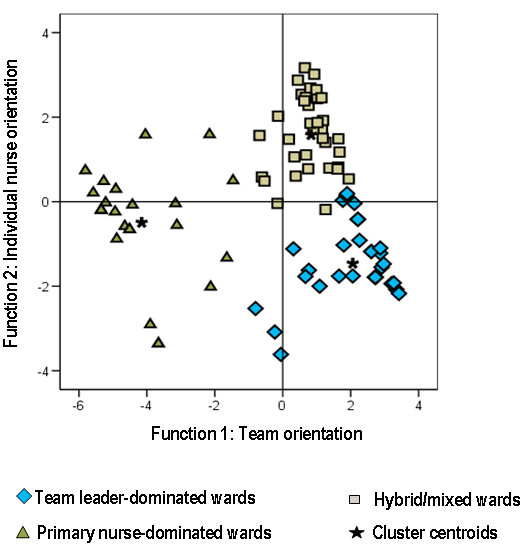
**Discriminant functions plot: three-cluster solution**.

In the discriminant function plot (Figure 2), the centroid of the TLD cluster had the highest value on function 1 (team orientation) and the lowest on function 2 (individual nurse orientation). The centroid of the PND cluster had its lowest value on function 1 (team orientation) and an intermediate value on function 2 (individual nurse orientation). The centroid of cluster C had an intermediate value on function 1 (team orientation) and the highest value on function 2 (individual nurse orientation). There was a continuous transition between TLD and cluster C.

**Figure 3:**
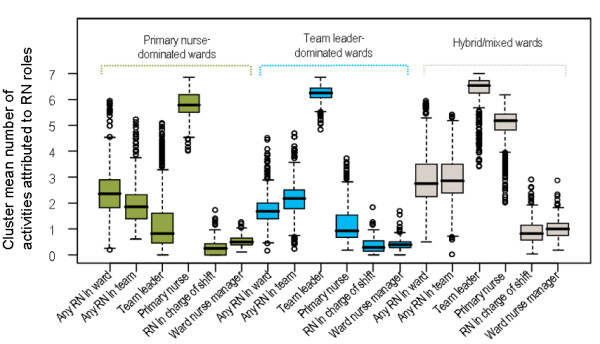
**Box plot of 400 bootstrap replications: three-cluster solution**.

Figure 3 presents the result from 400 bootstrap replications. In the bootstrap samples (which reflect the true underlying population variation), the TLD and PND clusters, observed in Figure 1, are stably reproduced. Cluster C scores were, on average, higher in the replications than in the sample on 'Any RN in the ward', 'Any RN in the patient's team', 'Nurse in charge of shift' and 'Ward nurse manager'. Consequently, cluster C was interpreted as containing wards intermediate between TLD and PND wards, as well as mixed wards with activities more evenly allocated to the RN roles, and named hybrid/mixed (HM). Further analysis showed that 94% of the bootstrap replications were consistent with this interpretation.

### Self-identification versus empirical classification

There was moderate correspondence between the empirical classification and the self-identification. The cross-tabulation showed that, for 78% of the wards empirically classified as TLD, the self-identification was 'Team or group nursing' and, for 74% of the PND wards, it was 'Primary nursing' or 'Modified primary nursing' (Table 2). In HM wards the self-identification varied, only one using a name that explicitly reflected the hybrid nature of the organizational model.

**Table 2:** Self-identification and external criteria by assigned cluster membership

**Assigned cluster membership**			**A: team leader dominated** **wards (*n *= 32)**		**B: primary nurse dominated** **wards (*n *= 23)**		**C: hybrid/mixed** **wards (*n *= 38)**		***p*^b^**		
				
			***n***	**%**	***n***	**%**	***n***	**%**	**A vs B**	**A vs C**	**B vs C**
									
Self-identification											
Primary or modified primary nursing			7	22	17	74	11	29	<0.001	0.589	0.001
Team or group nursing			25	78	6	26	23	61	<0.001	0.130	0.016
Combined team and primary nursing			0	0	0	0	1	3	-	1.000	1.000
Self-identification missing			0	0	0	0	3	8	-	0.245	0.284
Total			32	100	23	100	38	100			
									
Administration of oral medication^a^											
Team gives out to team's patients			30	94	6	26	35	92	<0.001	1.000	<0.001
Primary nurse gives out to her or his patients			3	9	17	74	5	13	<0.001	0.719	<0.001
									
Work allocation^a^											
Team leader allocates work			30	94	9	39	35	92	<0.001	1.000	<0.001
Each RN decides on care to her or his patients			6	19	12	52	15	40	0.018	0.072	0.427
									
Shift duty scheduling^a^											
Scheduling for each team			9	28	7	30	9	24	1.000	0.786	0.565
Scheduling to support patient-RN continuity			1	3	3	13	0	0	0.298	0.457	0.049
									
Patient allocation											
≥ 66% of patients are allocated to a team			24	75	19	86	27	71	0.493	0.791	0.219
≥ 66% of patients are allocated to a primary nurse			4	13	9	39	2	6	0.028	0.420	0.004

^a^The numbers of the two rows below do not add up to the *n *of the column header, as characteristics are confirmed by answering two separate questions, not mutually exclusive.

^b^Pairwise comparisons, Fisher's exact test

### Validation

In Table 2 we compared the clusters on supplementary data that had not been used in the cluster analysis. There was a significant difference between PND wards, on the one hand, and TLD and HM wards, on the other, in administration of oral medication (*p *< 0.001). Team leaders allocated the daily work on almost all of the TLD and HM wards, in contrast to the PND wards (*p *< 0.001). The allocation of 66% or more of patients to a primary nurse was practised in a significantly larger proportion of the PND compared with the HM wards. The differences in scheduling of shifts were not statistically significant. The supplementary variables did not discriminate between TLD and HM wards. The differences were the same when TLD and HM wards were merged, as in the two-cluster solution.

## Discussion

Through the use of cluster analysis for data reduction, we classified internal ward organization based on how the organizations functioned in practice. In a representative sample of wards in Norwegian general hospitals there was a moderate association between the empirical classification and the self-identification of organizational models. In many wards, the self-identification indicated a team or primary nursing organization, whereas the data suggested that they functioned in hybrid or mixed modes. This is an example of cluster analysis being used to categorize cases where no previous categories existed - often the case in healthcare organizations.

The results support previous warnings against the use of self-identification for nursing organizational models in research [3]. Outside the research field, in education as well as in discussions of nursing organization, we must be aware that terminology is an imperfect description of practice.

Differences between healthcare systems and between countries limit the generalizability of the classes identified in the current study, although the procedure used for classification is still generally applicable.

The three-cluster solution was consistent with the distribution of the questionnaire's supplementary variables, which were not used in constructing the clusters. These supplementary variables did not, however, differ between TLD and HM wards, which may be a reflection of the continuous transition between these two clusters, as observed in Figure 2, and also a consequence of limitations of the supplementary data, e.g. scheduling of shifts may not be a good variable for checking cluster consistency because choices may depend on, for example, night staffing more than on what is best from a strictly organizational point of view. By their design, the variables used in the clustering procedure give a more comprehensive description of the organization at the level of the patient-nurse interface.

In the current study, cluster analysis can be used to justify both the two- and the three-cluster solutions. Primary and team nursing organization are the main principles in ward organization, with local adjustments probably resulting in intermediate solutions. Over the last two decades, RNs have replaced practical nurses in many Norwegian hospitals. This means that teams now consist mainly of RNs, reducing some of the previous differences between team nursing and primary nursing, as observed in the HM cluster. The degree of separation between the clusters, as indicated by the discriminant functions plot, supported a three-cluster solution. The more detailed description of the three-cluster solution was regarded as more in line with theory and previous research and therefore more useful in practical research. However, the three classes of nursing organization were not identical to the generic types presented in the introduction.

In the study of Adams *et al*. [1], a similar clustering procedure identified three organizational models: in one cluster, labelled 'devolved nursing', responsibilities were mainly assigned to individual nurses, which corresponds to some degree with the PND wards in the current study. A second cluster, labelled 'two tier', was characterized by team work and a prominent role for the nurse manager. A third cluster was labelled 'centralized nursing', with less team work and more control in the hands of the ward nurse manager. The two-tier cluster has team work in common with our TLD wards, although it differs with regard to the role of the ward nurse manager, whose involvement in clinical practice has been reduced in Norway. This lack of responsibilities of the ward nurse manager on the TLD wards, and the absence of a parallel to the centralized cluster in the current study, can be explained by differences in time, settings and cultures.

The results of cluster analysis depend to some extent on the choices of the researcher, e.g. scaling of input variables, and should be viewed as an exploratory technique, which must eventually be validated by other means. We have demonstrated that our classification is stable with respect to sample variability and supported by validation from supplementary data. It is also consistent with theory and previous findings.

There is a possibility that the supplementary data were biased by the ward nurse managers wishing to present a consistent set of information [22]. Ideally, the classification should have been validated by detailed descriptions of the factual functioning of the ward organization by external informants, e.g. patients. Data collected from the patients in the current ward sample did not provide this possibility, but should be included in future use of the classification procedure. The possibility of obtaining validation data from outside the ward nursing services should also be considered, e.g. organization and workflow at hospital level.

The lack of pilot testing of the questionnaire is a limitation in the current study, but this limitation is mitigated by the use of items from previously developed questionnaires and by involvement of representatives of the survey population in the construction. Field observation studies and staff nurse surveys are potential alternatives to the approach used here, but these methods would require more resources. We consider a well-informed individual an adequate data source regarding global ward characteristics.

The classification that we obtained has been used to study the association of organizational models with RNs' ratings of quality of patient care, learning climate, job satisfaction and relationships with doctors in the RN survey data from the current ward sample [15]. Organizational models alone were not associated with RNs' ratings. When the models of analysis were expanded with explanatory variables describing other global ward characteristics, the association of these characteristics with the RNs' ratings varied with different organizational models. This supports the importance of including organizational models in studies of various aspects of nursing service management at the ward level.

## Conclusions

A meaningful and statistically valid classification of wards into three classes of nursing organizational models: team leader-dominated, primary nurse-dominated and hybrid/mixed models, was achieved through empirical cluster analysis. This was, however, only moderately consistent with the self-identification. The empirical classification is an objective approach to variable construction and can be generalized to the population of Norwegian hospitals, e.g. when comparing patient and quality-of-care outcomes across different organizational models. The actual classes cannot necessarily be generalized to other countries, but the method is generally applicable and could be developed to find a reliable and valid classification across both time and healthcare systems.

## Competing interests

The authors declare that they have no competing interests.

## Authors' contributions

ISS initiated the study, participated in the design, data collection, data analysis, and drafting and revisions of the manuscript. JH performed statistical analyses and interpretations and took part in the revisions of the manuscript. KS was involved in designing the study, drafting the paper and critically commenting on the manuscript. All authors read and approved the final manuscript.

## Pre-publication history

The pre-publication history for this paper can be accessed here:

http://www.biomedcentral.com/1472-6955/9/3/prepub

## Supplementary Material

Additional file 1 **Appendix 1**. Nurses' Questionnaire Click here for file

Additional file 2 **Appendix 2**. Discriminant function coefficients: three-cluster solution Click here for file
