# Supplementary material for: Classifying nursing organization in wards in Norwegian hospitals: self-identification versus observation
Source: BMC Nurs. 2010 Feb 9;9:3. doi: 10.1186/1472-6955-9-3 (PMC2832780; doi:10.1186/1472-6955-9-3)
Supplement: Additional file 1 — Appendix 1. Nurses' Questionnaire [file 1472-6955-9-3-S1.DOC]

# Appendix 1

| **A. Variables used in the cluster analysis** | | **Response options and coding** | |
| --- | --- | --- | --- |
| Number of activities usually performed, represented by scores from 0 to 7, by each of the below RN roles in the ward | |  | |
|  | a Any RN dealing with the patient |  | |
|  | b Any RN in patient’s team | Scores from 0 to 7 were coded for each RN role (a-f) by adding | |
|  | c Team leader | activities that were usually performed (Yes=1; No=0) | |
|  | d Primary nurse |  | -write and revise the nursing plan |
|  | e The RN in charge of shift |  | -report follow up in the nursing plan |
|  | f The ward nurse manager |  | -take part in the pre-round meeting with the doctors |
|  |  |  | -accompany doctors on rounds |
|  |  |  | -liaise with other professionals at hospital |
|  |  |  | -contact patients' relatives |
|  |  |  | -plan patients' discharge |
|  |  |  |  |
| **B. Variables used in consistency assessment** | |  | **Response options and coding** |
|  | How do you label the model of nursing service organization at the ward? |  | No fixed response option |
|  | How is oral medication administered on the ward? |  | -Each team gives out to the team’s patients (Yes=1; No=0) |
|  |  |  | -Each primary nurse gives out to her/his patients (Yes=1; No=0)  (more than one response option may apply) |
|  | Who allocates direct nursing tasks at the start of a shift? |  | -Team leader allocates tasks (Yes=1; No=0) |
|  |  |  | -Each RN decides on care to her/his patients (Yes=1; No=0)  (more than one response option may apply) |
|  | How are the shifts scheduled? |  | -It is done for each team (Yes=1; No=0) |
|  |  |  | -It is done to ease primary nurse practice (Yes=1; No=0)  (more than one response option may apply) |
|  |  |  |  |
|  | How many of the patients are allocated to a team? |  | None; 1 to 33%; 34 to 65%; 66 to 99%; and All (66% to All=1; Else=0) |
|  | How many of the patients are allocated to a primary nurse? |  | None; 1 to 33%; 34 to 65%; 66 to 99%; and All (66% to All=1; Else=0) |
