# Supplementary material for: Classifying nursing organization in wards in Norwegian hospitals: self-identification versus observation
Source: BMC Nurs. 2010 Feb 9;9:3. doi: 10.1186/1472-6955-9-3 (PMC2832780; doi:10.1186/1472-6955-9-3)
Supplement: Additional file 2 — Appendix 2. Discriminant function coefficients: three-cluster solution [file 1472-6955-9-3-S2.DOC]

# Appendix 2

## Discriminant function coefficients: three-cluster solution

|  |  | Functions | | |
| --- | --- | --- | --- | --- |
| Registered nurse (RN) roles |  | Team orientation |  | Individual nurse orientation |
|  |  |  |  |  |
| Any RN in ward |  | −0.174 |  | 0.350 |
| Any RN in the patient’s team |  | 0.290 |  | −0.160 |
| Team leader |  | 0.910 |  | 0.414 |
| Primary nurse |  | −0.482 |  | 0.919 |
| RN in charge of shift |  | −0.037 |  | 0.168 |
| Ward nurse manager |  | −0.085 |  | 0.104 |
|  |  |  |  |  |
